# Seasonal dynamics of myocardial infarctions in regions with different types of a climate: a meta-analysis

**DOI:** 10.1186/s43044-022-00322-5

**Published:** 2022-12-22

**Authors:** Nataliya V. Kuzmenko, Vitaliy A. Tsyrlin, Mikhail G. Pliss, Mikhail M. Galagudza

**Affiliations:** Almazov National Medical Research Centre, Saint Petersburg, Russia 197341

**Keywords:** Myocardial infarction, Season, Climate, Air temperature, Atmospheric pressure

## Abstract

**Background:**

It is known that cardiovascular events (CVE) occur more often in winter than in summer. However, dependence of myocardial infarction (MI) risk of on various meteorological factors is still not fully understood. Also, the dependence of the seasonal dynamics of MI on gender and age has not yet been studied. The purpose of our meta-analysis is to reveal dependence of the circannual dynamics of MI hospitalizations on gender, age, and characteristics of a region’s climate.

**Main body:**

Using Review Manager 5.3, we performed a meta-analysis of 26 publications on the seasonal dynamics of MI. In our meta-analysis, the relative MI risk was higher in colder compared to warmer seasons. Old age insignificantly increased the seasonal MI risk; gender did not affect the seasonal dynamics of MI, but MI was more common in men than in women. The severity of the seasonal dynamics of MI risk depended on the climate of the region. In a climate with a small amplitude of circannual fluctuations in air temperature, atmospheric pressure, and partial oxygen density in the air, as well as in regions where air humidity is higher in winter than in summer, an increase in MI risk in winter compared to summer was significant. It was not significant in regions with opposite climatic tendencies.

**Conclusions:**

Based on the results of our studies, it can be concluded that a decrease in air temperature increases in MI risk; in addition, hypoxia in the hot season can provoke CVE associated with ischemia.

**Supplementary Information:**

The online version contains supplementary material available at 10.1186/s43044-022-00322-5.

## Background

In 2019, approximately 18.6 million people died from cardiovascular diseases, of which 9.14 million deaths were due to ischemic heart disease [1]. Numerous studies have shown that cardiovascular events (CVE) are more common in winter than in summer [2]. This is facilitated by an increase in blood pressure, blood viscosity, and the level of circulating lipids in winter compared to summer [3–5].

The study [6] has demonstrated that weather stress is a trigger for 3.7% of all MI (myocardial infarction), but dependence of MI on various meteorological factors is still not fully understood. Thus, according to the results of the meta-analysis by Sun et al. [7], both increases and decreases in air temperature are associated with an increased MI risk. The Japanese study has shown an association of MI with heat [8]. However, no increase in MI cases was recorded in Moscow during an abnormally hot summer 2010, when during the month the daily air temperature averaged 33 °C with a maximum value of 37 °C [9]. In Tuscany (Italy) and northern France, an increase in hospital admissions for MI was observed with a decrease in air temperature [10, 11]. Danet et al. [10], according on the results of 10 years of observations, noted a V-shaped relationship between atmospheric pressure and MI. Research in Texas has established an association between atmospheric pressure drop and MI [12]. In other studies, on the contrary, MI was more frequent in weather with increased atmospheric pressure [13]. Also, these authors noted the association of an increase in MI risk with high air humidity. Thus, the results of clinical trials are highly controversial.

The study by Cannistraci et al. [14] showed that MI risk is minimal in summer in regions with different climate (Finland, Italy, Scotland, China, Japan, Australia). However, this study is limited by a small number of MI cases and a short follow-up period. To the best of our knowledge, no one has studied the seasonal dynamics of MI depending on the vector and amplitude of annual meteorological factor variations.

It is known that MI occurs more often in men than in women, and MI risk increases with age [15]. In addition, it has been shown that the effect of external and internal triggers on MI risk depends on gender and age [6, 15]. Some studies have shown that association of MI with weather conditions is most pronounced in elderly people [10, 16]. However, so far no one has studied the effect of gender and age on the seasonal dynamics of MI risk.

The purpose of our meta-analysis is to reveal dependence of the circannual dynamics of MI hospitalizations on gender, age and characteristics of a region’s climate. Elucidation of the relationship between MI risk and fluctuations in weather conditions is an important task for the prevention of MI and mortality from them.

## Main text

### Methods

A systematic review was conducted following the Meta-analysis of Observational Studies in Epidemiology guidelines [17]. The search for publications was carried out independently by two researchers. Two authors independently evaluated all records by title, abstract, or full text for potentially eligible studies, and any disagreement was resolved by consensus.

The search strategies were based on combinations of keywords related to climate (season, climate, weather, winter, summer, spring, autumn, fall, temperature, heat, hot, warm, cold, atmospheric pressure, barometric pressure, air humidity, relative humidity) and MI (myocardial infarction, ischemic attack, ischemic heart disease, myocardial ischemia, cardiac ischemia, coronary heart disease, heart disease, vascular disease, cardiovascular diseases) and hospitalization (morbidity, hospitalization, event, hospital admissions, incident, case, risk) and gender/sex (man, woman, male, female) and age (young, elderly, age, old). The “Human” filter has been applied. The search was carried out in the databases of PubMed, Scopus, Russian database eLibrary, Google Scholar with a limited publication period of 1940–2021 yy due to imperfections in the diagnostic methods in early studies. The search was carried out in English and Russian. We also searched the reference lists of the included studies.

We selected publications on the seasonal dynamics of hospitalizations for MI, but no deaths from MI. The method of displaying statistical data on the paper was supposed to enable us to conduct a meta-analysis. For example, articles in which the number of hospitalizations was presented per 100,000 population or week, or was expressed only as odds ratio, or as regression without indicating absolute values were excluded. In the selected studies for this meta-analysis, data for four seasons (winter, spring, summer, and autumn) had to be presented. Regions with mountainous climate were not included in the meta-analysis. If a group of authors published several articles on the same topic, the publication with data presented for the maximum observation period was selected.

Only high- and medium-quality studies were selected for meta-analysis. When assessing the quality of publications, the following were taken into account: diagnostic methods, duration of observation, study localization, presentation of seasons, presentation of meteorological data, sample size, gender / age representation and presentation of results.

Two researchers independently searched for data. After reconciliation, the data were included in the meta-analysis. When conducting a meta-analysis, data of MI cases per season and the total number MI was extracted or calculated from publications. The data were processed without regard to gender and age (data for gender and age groups were pooled), as well as taking into account gender and age. To study the seasonal dynamics of MI risk by gender and age, only those publications were selected in which data were presented separately for gender / age groups. 

If the study accurately indicated the localization of the study region, then the publication was included in the meta-analysis, which assessed dependence of MI risk on the characteristics of the climate of the region. If the article presented meteorological data, then we used them. Otherwise, using the archived data, we calculated the meteorological parameters (temperature and relative humidity of air, atmospheric pressure, partial density of oxygen in the air (PO_2_)), as described in the work [18]. Depending on annual fluctuations in a meteorological factor in a region of a study, publications were divided into two subgroups: one with the maximum amplitude of change and another with the minimum amplitude of change of a meteorological factor.

We used the statistical program Review Manager 5.3 for meta-analysis. Mantel–Haenszel (odds ratio) test was applied to assess MI risk. Study heterogeneity included in the meta-analysis was determined using criterion I^2^. The choice of fixed-effects or randomized-effects model was carried out according to the recommendations of Borenstein et al. [19]. A Z-test was used to assess the statistical significance of the total results. The confidence interval was 95%, and differences were considered statistically significant at *p* < 0.05. Funnel plots, Egger’s test, and Begg’s test were used to detect publication bias. Egger’s test and Begg’s test were calculated using MedCalc program.

### Results

**Fig. 1:**
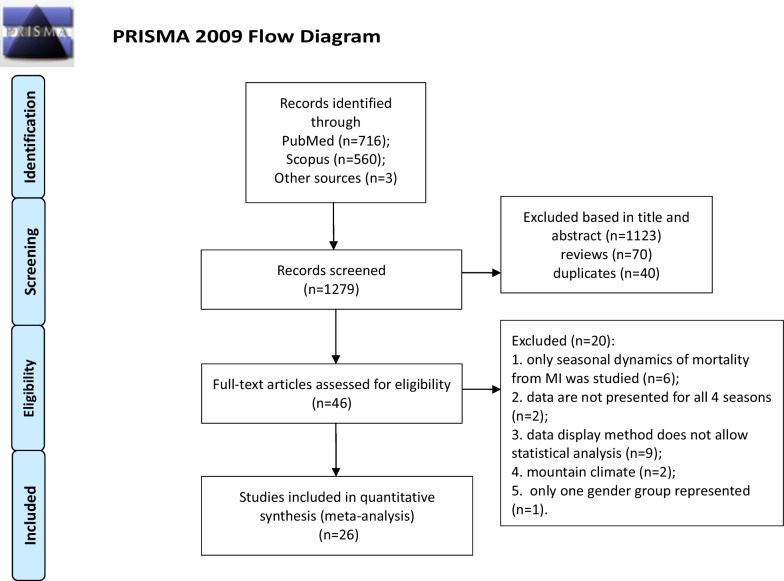
Flow diagram according to the preferred reporting items for systematic reviews and meta-analysis guidelines (http://prisma-statement.org/)

A total of 1279 publications were found. We selected 26 publications reporting the seasonal dynamics of MI [8, 9, 11, 13, 16, 20–40] (Fig. 1). We selected only medium- to high-quality studies (Table 1 and Additional file 1: Table S1). Table 1 and Additional file 1: Table S1 show the main characteristics of the publications included in our meta-analysis. Some studies were not included in our meta-analysis for various reasons (Fig. 1, Additional file 1: Table S2). Assessment of funnel plots, Egger’s test, and Begg’s test showed no evidence of publication bias (Additional file 1: Figs. S1 and S2).

**Table 1:** Selected publications devoted to the study of seasonal dynamics of MI

Publications	Period of the study (yy)	Location of the study, geographical coordinates, altitude	Total number of MI	Age (years)	Sex, male (%)	Diagnostics
Akioka [8]	2012–2013	Oita, Japan, 33º14´N, 131º36´ E, 9 m a.s.l	186	71	76.3	–
Depasquale [20]	1952–1955	New Orleans, USA, 29º58´N, 90º06´ W, 4 m a.s.l	1582	–	–	ECG
Didier [13]	2009–2016	Brest, France, 48°23´ N, 4° 29´W, 34 m a.s.l	742	62.4	71.3	ECG
Douglas [21]	1962–1971	Scotland	125,799	65*	64.5**	–
González Hernández [22]	1995–1999	Valencia, Spain, 39°28´ N, 00° 22´W, 15 m a.s.l	8329	65*	76	–
Heyer [23]	1946–1951	Dallas, USA, 32º46´N, 96º47´ W, 131 m a.s.l	1386	–	–	ECG
Hong [24]	1997–2006	South Korea	265,935	65*	59.9**	–
Khan [25]	2005–2015	Germany	3,008,188	70*	62.3**	WHOC
Keller 2014 [26]	2010–2012	Dhaka, Bangladesh, 23º42´N, 90º22´ E, 60 m a.s.l	2374	–	–	WHOC
Kozlovskaia [9]	2009–2012	Moscow, Russia, 55º45´N, 37º37´ E, 200 m a.s.l	63,412	≥ 40	53	ECG
Ku [27]	1992–1996	Kaohsiung City, Taiwan, 22º38´N, 120º16´ E, 9 m a.s.l	540	62.3	74.1	ECG, CK
Lashari [28]	2011	Karachi, Pakistan, 24º51´N, 67º E, 8 m a.s.l	428	48.5	61	ECG, CK, T
Lin [29]	2005–2016	Taipei, Taiwan, 25° N 121º 32´ E, 9 m a.s.l	2151	59	83	–
Mahajan [30]	2007–2014	USA	321,888	60	65**	–
Manfredini [31]	1998–2006	Ferrara, Italy, 44º50´N, 11º37´ E, 9 m a.s.l	64,191	70	62.9**	WHOC
Mintz [32]	1940–1945	Chicago, USA, 41º54´N, 87º39´ W, 200 m a.s.l	572	59.5	68.5	ECG
Morabito [11]	1998–2002	Florence, Italy, 43º47´N, 11º15´ E, 50 m a.s.l	2683	65*	66**	WHOC
Moschos [33]	1988–1998	Rhodes, Greece, 36º10´N, 28º E, 11 m a.s.l	1196	–	75	ECG, CK
Nagarajan [34]	2003–2008	USA	82,971	67.5	60.1**	-
Park [35]	2007–2016	Seoul, South Korea, 37º35´N, 127º E, 38 m a.s.l	279	59.2	80.7**	CMR, CK
Radišauskas [16]	1995–2007	Kaunas, Lithuania, 54º54´N, 23º56´ E, 47 m a.s.l	6753	44.5*	57.7**	WHOC
Rumana [36]	1988–2003	Takashima, Japan, 35º21´N, 136º E, 94 m a.s.l	335	71.7	64.8	WHOC
Sharif Nia [37]	2013–2015	Sari, Iran, 36°33´ N 53º E, 32 m a.s.l	6377	–	–	ECG, CK, T
Spencer [38]	1994–1996	USA	124,239	65.7*	64**	–
Spielberg [39]	1980–1988	Dessau, Germany, 51º50´N, 12º14´ E, 60 m a.s.l	2906	67.8	60.1	WHOC
Thakur [40]	1979–1983	Patna, India, 25º37´N, 85º08´ E, 53 m a.s.l	1217	–	–	ECG, CK

*WHOC* World Health Organization Criteria, *ECG* Electrocardiography, *CK* Creatine kinase, *T* Troponin, *CMR* Cardiovascular magnetic resonance

* is statistics presented separately for groups of different ages, ** is statistics presented separately for men and women, and (-) is no information

We studied the seasonal dynamics of MI in 20 cities. Cities were located in various climatic zones, ranging from temperate to tropical (Table 2). The climate of the regions differed in the amplitude of changes from winter to summer in air temperature, magnitude/variability of atmospheric pressure, and PO_2_ (Table 2). In Dhaka, Karachi, Brest (France), the differences in average temperatures between winter and summer do not exceed 10 °C; in Moscow, Chicago, Seoul, the differences are more than 23 °C. Circannual fluctuations of PO_2_ in Valencia, Dhaka, Brest (France) do not exceed 16 g/m^3^; in Seoul, Takashima, Moscow, Chicago, they are more than 28 g/m^3^. Atmospheric pressure is usually higher and more variable in winter than in summer. For the climate of the temperate zone of Europe, pronounced seasonal dynamics of atmospheric pressure variability, but not its average value, are characteristic. On the contrary, in the regions of East Asia and the Middle East, atmospheric pressure is 10–20 hPa higher in winter than in summer, while the seasonal dynamics of atmospheric pressure variability is weakly expressed. Relative humidity usually varies from winter to summer. In Europe, relative humidity is higher in winter than in summer, while in East Asia, its annual trend is reversed.

**Table 2:** Seasonal dynamics of meteorological factors in the studied regions (regions are arranged in decreasing order of geographical latitude)

Regions	Air temperature °C		Atmospheric pressure hPa		Atmospheric pressure variability hPa		Relative Humidity %		PO_2_ g/m^3^	
	Winter	Summer	Winter	Summer	Winter	Summer	Winter	Summer	Winter	Summer
*Europe*										
Moscow, Russia	− 7.2	19.6	997	993	11.7	5	88	77	301	272
Kaunas, Lithuania	− 2.7	18.2	1001	1001	11.6	5.5	90	75	298	274
Dessau, Germany	2.9	21.6	1001	1000	9.2	4.8	89	72	291	270
Brest, France	7.5	16.6	1006	1004	9.6	5.6	88	85	287	276
Ferrara, Italy	3	23	1012	1007	8.5	3.8	82	66	295	270
Florence, Italy	8.2	26.5	1011	1006	8.1	3.2	77	67	288	265
Valencia, Spain	12.8	25.8	1011	1012	61	67	7.9	3.4	283	268
Rhodes, Greece	14.0	26.9	1012	1003	5.3	2.4	76	64	281	264
*Asia*										
Seoul, South Korea	− 0.2	24.2	1010	994	4.9	3.9	54	72	296	262
Sari, Iran	10.4	29.2	1017	1005	7.0	4.0	58	66	288	262
Takashima, Japan	4	25	1015	1004	5.6	4.1	68	70	295	266
Oita, Japan	8.8	24.7	1020	1007	4.9	3.8	65	78	291	267
Patna, India	19.6	31	1004	989	2.5	2.2	75	80	273	253
Taipei, Taiwan	15.1	27.7	1016	1001	4.0	4.0	83	78	281	261
Karachi, Pakistan	20.8	29.6	1009	994	2.7	2.3	50	73	274	257
Dhaka, Bangladesh	19.8	29.3	1008	998	2.7	2.7	67	84	274	258
Gayusan, Taiwan	16	27.6	1013	1002	3.2	3.5	83	78	279	262
*North America*										
Chicago, USA	− 0.3	24.8	990	988	8.1	4.4	73	69	292	262
Dallas, USA	6.9	28.1	995	989	6.8	2.8	66	62	286	259
New Orleans, USA	14.3	28.6	1016	1011	5.7	2.6	86	90	282	262

We analyzed a total of 4,096,659 MI cases. The meta-analysis of the seasonal dynamics of MI showed that MI more often occurs in winter than in summer and autumn (*p* < 0.05). Of all MI cases per year, accounting for winter 26.3 ± 2.7%, summer 23.7 ± 2.5%, spring 25.4 ± 2.6%, and autumn 24.7 ± 2.3%. The relative risk of MI was higher in the colder season than in the warmer one (Table 3). The research results were highly heterogeneous. This can be explained by different lengths of an observation period, as well as differences in climatic and socio-economic conditions.

**Table 3:** Dependence of myocardial infarction risk on the season

Compared seasons		Number of studies	Total	Odds ratio	*I*^2^%	Overall effect test	
Season 1 / total	Season 2 / total					Z	*P*
*All*							
Winter / 1,055,200	Summer / 969,524	26	4,096,659	1.16 [1.08, 1.25]	99	4.17	0.0001
Winter / 1,055,200	Spring / 1,047,486	25	4,089,906	1.04 [0.98, 1.10]	99	1.18	0.24
Winter / 1,055,200	Autumn / 1,023,992	25	4,089,906	1.07 [1.03, 1.11]	97	3.63	0.0003
Autumn / 1,023,992	Summer / 969,524	25	4,089,906	1.08 [1.03, 1.14]	99	3.11	0.002
Spring / 1,047,486	Summer / 969,524	25	4,089,906	1.13 [1.09, 1.18]	98	6.24	0.00001
Spring / 1,047,486	Autumn / 1,023,992	25	4,089,906	1.04 [1.00, 1.08]	98	1.97	0.05
*Men*							
Winter / 637,493	Summer / 593,271	10	2,494,030	1.14 [1.03, 1.26]	100	2.60	0.009
Winter / 637,493	Spring /639818	10	2,494,030	1.03 [0.97, 1.10]	99	0.93	0.35
Winter / 637,493	Autumn / 623,448	10	2,494,030	1.03 [0.99, 1.06]	96	1.52	0.13
Autumn / 623,448	Summer / 593,271	10	2,494,030	1.12 [1.03, 1.21]	99	2.75	0.006
Spring / 639,818	Summer / 593,271	10	2,494,030	1.10 [1.05, 1.16]	98	3.66	0.0003
Spring / 639,818	Autumn / 23,448	10	2,494,030	0.99 [0.94, 1.04]	98	0.41	0.68
*Women*							
Winter / 388,686	Summer / 353,702	10	1,502,478	1.21 [1.10, 1.34]	99	3.91	0.0001
Winter / 388,686	Spring / 382,943	10	1,502,478	1.07 [1.00, 1.15]	98	1.87	0.06
Winter / 388,686	Autumn / 377,147	10	1,502,478	1.03 [0.98, 1.07]	96	1.06	0.29
Autumn / 377,147	Summer / 353,702	10	1,502,478	1.18 [1.10, 1.26]	98	4.49	0.00001
Spring / 382,943	Summer / 353,702	10	1,502,478	1.11 [1.06, 1.17]	97	4.02	0.0001
Spring / 382,943	Autumn / 377,147	10	1,502,478	0.96 [0.90, 1.01]	98	1.53	0.13
*Young and middle-aged people*							
Winter / 396,959	Summer / 375,442	7	1,562,568	1.19 [1.03, 1.37]	100	2.34	0.02
Winter / 396,959	Spring / 402,294	7	1,562,568	1.02 [0.92, 1.12]	99	0.32	0.75
Winter / 396,959	Autumn / 387,873	7	1,562,568	1.03 [0.97, 1.08]	96	0.99	0.32
Autumn / 387,873	Summer / 375,442	7	1,562,568	1.15 [1.03, 1.29]	99	2.43	0.02
Spring / 402,294	Summer / 375,442	7	1,562,568	1.13 [1.06, 1.20]	97	3.73	0.0002
Spring / 402,294	Autumn / 387,873	7	1,562,568	1.00 [0.93, 1.08]	98	0.04	0.97
*Old people (*≥ *65 years old)*							
Winter / 514,066	Summer / 457,247	7	1,972,942	1.30 [1.14, 1.48]	100	3.85	0.0001
Winter / 514,066	Spring / 505,756	7	1,972,942	1.08 [0.97, 1.21]	99	1.42	0.16
Winter / 514,066	Autumn / 495,873	7	1,972,942	1.07 [1.02, 1.11]	94	3.02	0.003
Autumn / 495,873	Summer / 457,247	7	1,972,942	1.22 [1.09, 1.36]	99	3.50	0.0005
Spring / 505,756	Summer / 457,247	7	1,972,942	1.17 [1.12, 1.22]	94	7.21	0.00001
Spring / 505,756	Autumn / 495,873	7	1,972,942	0.98 [0.91, 1.06]	99	0.44	0.66

In all seasons, MI was more common in men (67%) than in women (33%), in old people (≥ 65 years old) than in young and middle-aged people (Table 3). Gender did not significantly affect the seasonal dynamics of MI risk (Table 3). According to the results of 7 studies, old age insignificantly increased MI risk in the cold season compared to the warmer one (Table 3).

**Fig. 2:**
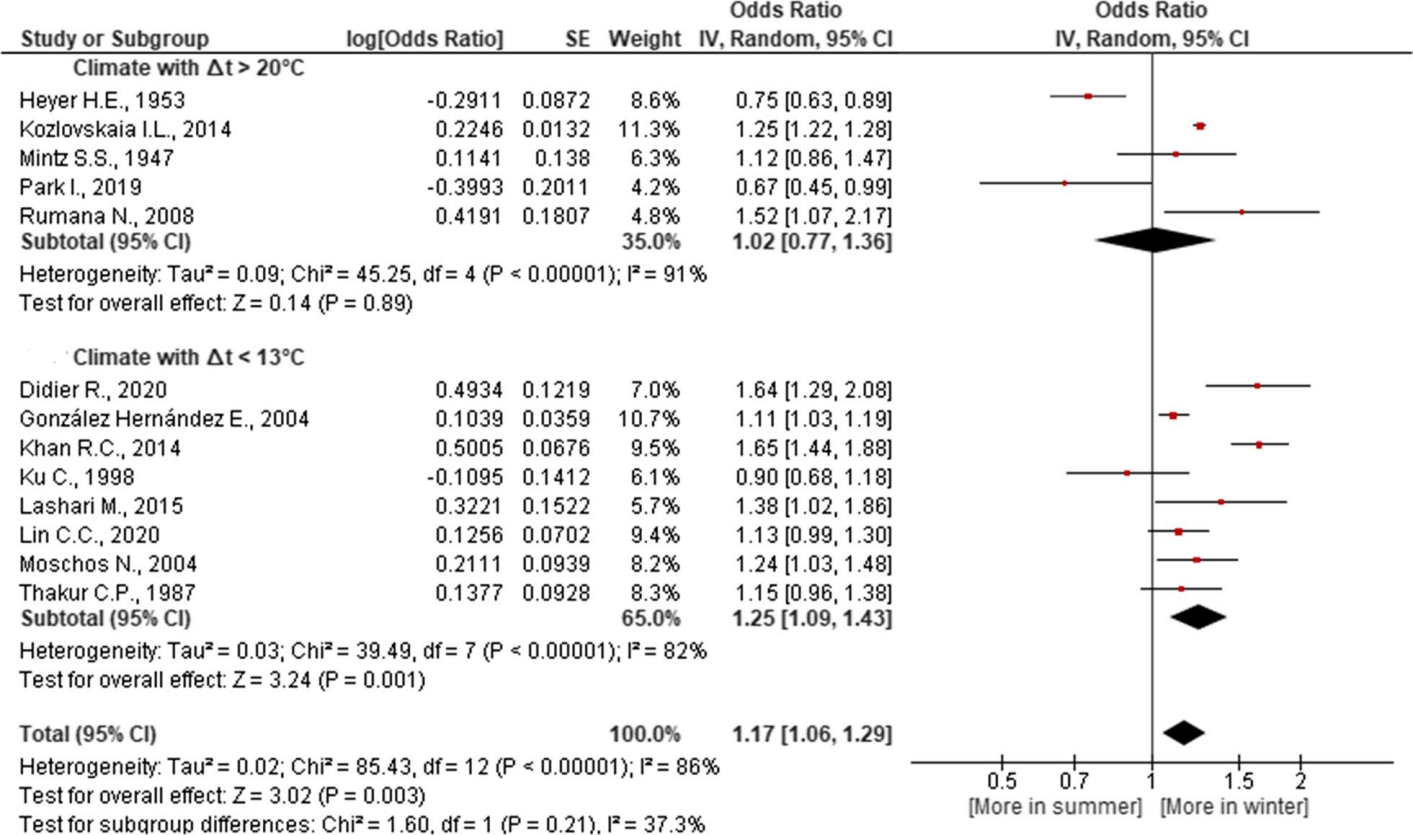
Association of seasonal risk of myocardial infarction with air temperature

**Fig. 3:**
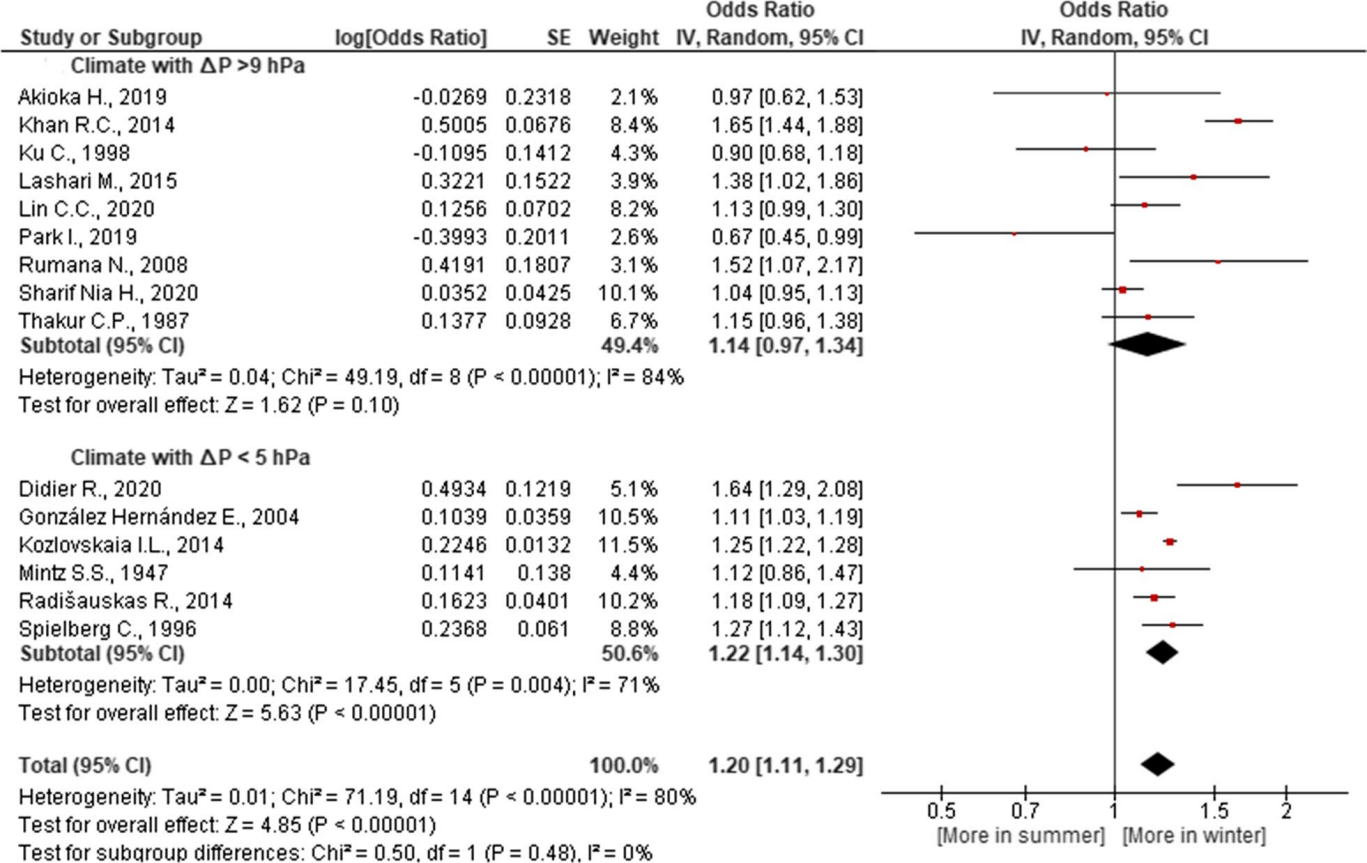
Association of seasonal risk of myocardial infarction with atmospheric pressure

**Fig. 4:**
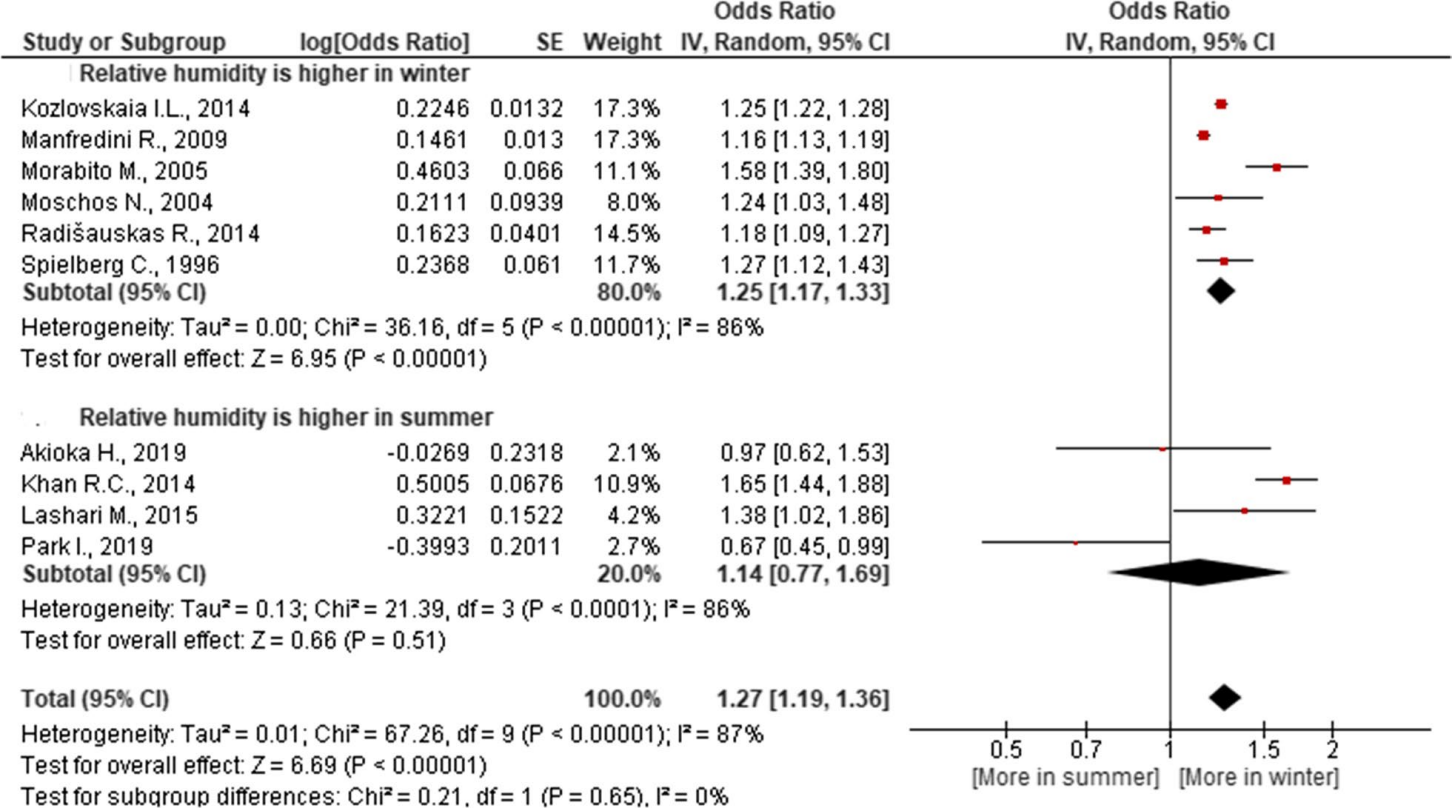
Association of seasonal risk of myocardial infarction with relative humidity

**Fig. 5:**
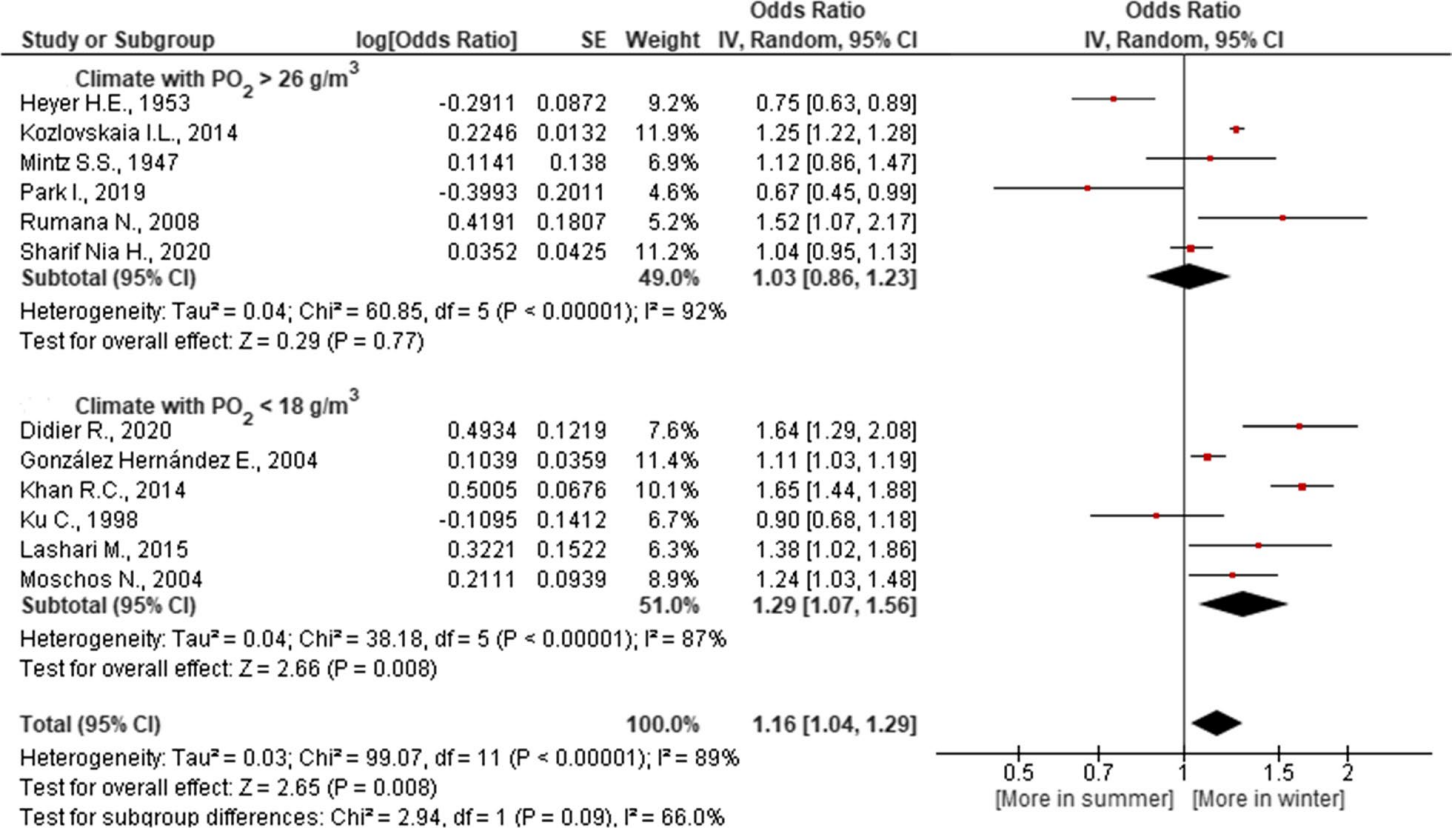
Association of seasonal risk of myocardial infarction with partial density of oxygen in air

According to the results of our meta-analysis, the severity of the seasonal dynamics of MI risk depended on the climate of the region. In a climate with a small amplitude of annual fluctuations in air temperature, atmospheric pressure and PO_2_, as well as in regions where relative humidity is higher in winter compared to summer, an increase in MI risk in winter compared to summer was significant. It was not significant in regions with opposite climatic tendencies (Figs. 2, 3, 4 and 5). Studies [21, 24, 25, 30, 34, 38] were not included in the meta-analysis of dependence of MI on the regional climate, since they present statistics for the whole country, and not for a specific region (Table 1).

### Discussion

This meta-analysis revealed that MI was more likely to occur during colder seasons than in warmer seasons, and the highest MI incidence was observed in winter. The results of our meta-analysis are consistent with the results of clinical studies in which an association of an increase in MI risk with a decrease in air temperature was observed in various climatic zones [7, 40–44]. However, seasonal MI risk was increased in regions with small differences between winter and summer temperatures. Perhaps this is due to the fact that in regions with warm winter, central heating is often absent, and for this reason, people feel better seasonal fluctuations in air temperature. In addition, besides air temperature, other meteorological factors (atmospheric pressure, relative humidity) can also influence seasonal dynamics of MI. A similar association between blood pressure and air temperature was previously reported [3, 45]. Thus, according to the results of the meta-analysis [45], in people blood pressure was higher in the cold season compared to the warmer one, and the maximum increase in blood pressure in winter was observed in regions with lower annual fluctuations in air temperature. It confirms dependence of MI risk on an increase in blood pressure. In addition, it has been shown that a decrease in air temperature stimulates an increase in hematocrit and cholesterol levels, the formation of cholesterol plaques, and their rupture [4, 5, 43]. Shibuya et al. [46] report the worst angiographic parameters (minimum vessel lumen, maximum lipid plaque) in patients with acute coronary syndrome in winter.

It is known that old age increases the reactivity of the sympathetic nervous system and blood pressure to cold [3, 47]. In addition, with aging, visceral vasodilation (in particular, of the coronary vessels) decreases with cooling [48]. According to our meta-analysis, old age was associated with a growth of MI cases in all seasons. Besides, in the cold season, MI risk increased slightly in old people compared to younger people. Gender did not affect the seasonal dynamics of MI risk, but MI was more common in men than in women. It is generally believed that low MI risk in women is due to the protective effects of female sex hormones, but differences in lifestyle (diet, smoking, alcohol) may also be important [15]. Other authors also reported that male gender and old age increased MI risk in cold weather [10, 11, 41, 42, 44, 49]. Moreover, Hong et al. and Park et al. [24, 35] observed that male gender increases MI risk in the heat. In addition, the authors [24] noted a growth of MI events during summer only in people younger than 65 years old.

Similar to MI, IS (ischemic stroke) can be provoked by fluctuations in blood pressure and blockage of the vessel by thrombus or cholesterol plaque. Previously, it was shown that the seasonal dynamics of IS depend on the climate of the region. A drop in atmospheric pressure and high relative humidity during hot weather were associated with an increased IS risk in summer compared to winter. At the same time, weakly pronounced seasonal dynamics of the average monthly atmospheric pressure and high air humidity in winter are associated with a slight increase in IS risk in winter [50]. It is known that atmospheric pressure and relative humidity determine PO_2_ [51], the partial pressure of alveolar oxygen [52], and the oxygen saturation of hemoglobin [53, 54]. Meteorological factors, associated with a decrease in PO_2_ and a shift in IS risk for the summer, weakened the severity of the seasonal dynamics of MI but usually did not change the vector of seasonal fluctuations of MI to the opposite. Nevertheless, studies of the relationship of CVE to decreased oxygen saturation of hemoglobin, for example, in apnea or in mountain climates, have shown an increased risk for both IS and MI [55–58]. On the other hand, oxygen therapy was found to be more effective in preventing IS than MI [59, 60]. Probably for this reason, in the case of MI, an increase in PO_2_ in winter does not always compensate for the negative effect of a winter increase in blood pressure and lipid profile parameter [3, 5].

Some authors have associated an increase in CVE risk in hot weather with an increase in blood viscosity during hyperthermia [61, 62]. It is known that high hematocrit can increase the risk of IS and MI by stimulating thrombus formation [63–65]. Although elevated sweating during heat contributes to hemoconcentration, this may be offset by increased water intake. Besides, in the Brazilian study, an association of an increase in thromboembolism incidents with a decrease in air temperature was revealed [66]. Moreover, the meta-analysis [4] showed that hematocrit in humans is higher in winter than in summer, and in regions where atmospheric pressure is significantly lower in summer compared to winter, summer decrease in hematocrit is maximal. There are observations that the initial stage of hypoxia is characterized by a slight decrease in hematocrit due to a lessening in the corpuscular volume of erythrocytes [67]. It has been found that an increase in hematocrit within the normal range improves the supply of oxygen to organs [68, 69].

It is known that atmospheric pressure drop is also associated with the risk of thrombus formation. A 10 hPa decrease in atmospheric pressure has been shown to increase the relative risk of deep vein thrombosis by 2.1% [70]. There is an observation that hypobaric exposure activates coagulation [71]. However, based on the results of publications [72–75] included in the meta-analysis Zhao et al. [76], investigating the seasonal dynamics of thromboembolism, it can be concluded that in regions with low atmospheric pressure in summer (China, Korea), thromboembolism occurs more often in winter than in summer. It was shown that in addition to cholesterol and hematocrit, levels of blood coagulation factors increase in winter compared to summer [77–79]. As a result, summer drop in atmospheric pressure, most likely, increases the risk of CVE associated with ischemia, not activating thrombus formation, but causing a decline in PO_2_, which decreases even more by heat and high humidity. It should be noted that high relative humidity in winter can increase the feeling of cold and promote CVE risk.

The regularities of the effect of circannual fluctuations in atmospheric pressure on the seasonal dynamics of CVE associated with ischemia that we discovered allow us to explain the contradictions between the results of clinical observations carried out in different climatic zones. For example, in Oita and Seoul, where in summer compared to winter, atmospheric pressure drops significantly and air humidity increases, heat provokes MI [8, 35]. At the same time, in Moscow and Florence, hot weather is not usually accompanied by atmospheric pressure drop and not associated with an increase in MI events [9, 11].

## Conclusions

In our meta-analysis, the relative MI risk was higher in colder compared to warmer seasons. Based on the literature data, it is obvious that the seasonal MI risk is associated with circannual changes in biochemical and physiological parameters (Table 4). Old age insignificantly increased the seasonal MI risk; gender did not affect the seasonal dynamics of MI, but MI was more common in men than in women. The severity of the seasonal dynamics of MI risk depended on the climate of the region (Table 4). In a climate with a small amplitude of circannual fluctuations in air temperature, atmospheric pressure, and partial oxygen density in the air, as well as in regions where air humidity is higher in winter than in summer, an increase in MI risk in winter compared to summer was significant. It was not significant in regions with opposite climatic tendencies (Table 4). Based on the results of our studies, it can be concluded that a decrease in air temperature increases in MI risk, in addition, hypoxia in the hot season can provoke CVE associated with ischemia.

**Table 4:** Factors determining the seasonal risk of myocardial infarction

Winter	Summer
Seasonal changes in biochemical and physiological parameters: Increase in blood pressure Increase in lipid profile parameters Increase in hematocrit Increase in thrombosis	Seasonal changes in biochemical and physiological parameters: Decrease in hematocrit Decrease in blood oxygen saturation
Meteorological factors: Low air temperature Small amplitude of annual fluctuations in air temperature High relative humidity Small amplitude of annual fluctuations in atmospheric pressure Small amplitude of annual fluctuations in PO_2_	Meteorological factors: High air temperature Large amplitude of annual fluctuations in air temperature High relative humidity Significant drop in atmospheric pressure Large amplitude of annual fluctuations in atmospheric pressure Significant drop in PO_2_ Large amplitude of annual fluctuations in PO_2_
Other factors: Male gender Old age	Other factors: Male gender Old age

## Limitation

The limitation of the meta-analysis that affects the final result may be such factors as different length of the observation period in different publications, different sample size when comparing subgroups, high heterogeneity of results. In addition, in the absence of meteorological data regarding the period of a study, we considered a period of 10 years. This may have led to insufficient accuracy in the processing of meteorological data in such cases. However, it is known that seasonal trends in meteorological factors are quite stable within climatic zones. In this meta-analysis, when studying MI risk from a climate of a region, we compared only two seasons, winter and summer, since the meteorological conditions in these seasons are as stable and contrasting as possible. It is rather difficult to take into account the peculiarities of weather conditions in spring and autumn in a meta-analysis, but it is possible in clinical observations.

We did not take into account lifestyle factors (diet, alcohol consumption, physical activity, holidays, fasts) and outbreaks of respiratory viral infections, which may be important for MI risk [33, 80, 81]. However, studies of seasonal MI risk during the COVID-19 pandemic (2020–2022) were not included in our meta-analysis.

## Supplementary Information

**Additional file 1**. Supplemental material including figures S1–S2 and tables S1–S2.

## Data Availability

The data and materials are presented in the publication. In addition, Supplementary Materials are available.

## Abbreviations

CVE: Cardiovascular events
MI: Myocardial infarction
HS: Hemorrhagic stroke
IS: Ischemic stroke
PO_2_: Partial density of oxygen in the air

## References

[1] Roth GA, Mensah GA, Johnson CO et al., GBD-NHLBI-JACC Global Burden of Cardiovascular Diseases Writing Group. (2020) Global burden of cardiovascular diseases and risk factors, 1990–2019: update from the GBD 2019 study. J Am Coll Cardiol 76(25):2982–3021. doi: 10.1016/j.jacc.2020.11.010. Erratum in: J Am Coll Cardiol. 2021 20;77(15):1958–1959. 10.1016/j.jacc.2020.11.010 PMC7755038 33309175

[2] Marti-Soler H, Gubelmann C, Aeschbacher S, Alves L, Bobak M, Bongard V, Clays E, de Gaetano G, Di Castelnuovo A, Elosua R (2014). Seasonality of cardiovascularrisk factors: an analysis including over 230 000 participants in 15 countries. Heart.

[3] Kollias A, Kyriakoulis KG, Stambolliu E, Ntineri A, Anagnostopoulos I, Stergiou GS (2020). Seasonal blood pressure variation assessed by different measurement methods: systematic review and meta-analysis. J Hypertens.

[4] Kuzmenko NV, Tsyrlin VA, Pliss MG (2021). Seasonal dynamics of red blood parameters in healthy people in regions with different types of climate: a meta-analysis. Izv Atmos Ocean Phys.

[5] Ma X, Yan H, Zhang H, Wang M, Zhang Q, Zhou X (2020). Progress in the seasonal variations of blood lipids: a mini-review. Lipids Health Dis.

[6] Culić V, Eterović D, Mirić D (2005). Meta-analysis of possible external triggers of acute myocardial infarction. Int J Cardiol.

[7] Sun Z, Chen C, Xu D, Li T (2018). Effects of ambient temperature on myocardial infarction: a systematic review and meta-analysis. Environ Pollut.

[8] Akioka H, Yufu K, Teshima Y, Kawano K, Ishii Y, Abe I, Kondo H, Saito S, Fukui A, Okada N, Nagano Y, Shinohara T, Nakagawa M, Hara M, Takahashi N (2019). Seasonal variations of weather conditions on acute myocardial infarction onset: Oita AMI registry. Heart Vessels.

[9] Kozlovskaia IL, Bulkina OS, Lopukhova VV, Kolmakova TE, Karpov IuA, Starostin IV, Baratashvili VL, Rubinshtein KG, Emelina SV, Borovikov VP (2014). Trends in hospitalizations of patients with acute coronary syndrome and indicators of the atmospheric state in Moscow in 2009–2012. Ter Arkh.

[10] Danet S, Richard F, Montaye M, Beauchant S, Lemaire B, Graux C, Cottel D, Marécaux N, Amouyel P (1999). Unhealthy effects of atmospheric temperature and pressure on the occurrence of myocardial infarction and coronary deaths. A 10-year survey: the Lille-World Health Organization MONICA project (Monitoring trends and determinants in cardiovascular disease). Circulation.

[11] Morabito M, Modesti PA, Cecchi L, Crisci A, Orlandini S, Maracchi G, Gensini GF (2005). Relationships between weather and myocardial infarction: a biometeorological approach. Int J Cardiol.

[12] Houck PD, Lethen JE, Riggs MW, Gantt DS, Dehmer GJ (2005). Relation of atmospheric pressure changes and the occurrences of acute myocardial infarction and stroke. Am J Cardiol.

[13] Didier R, Le Ven F, Ouchiha M, Nicol PP, Auffret V, Oueslati C, Nasr B, Jobic Y, Noel A, Aidonidis M, Koifman E, Mansourati J, Gilard M (2020). Analysis of weather exposure 7 days before occurrence of ST-segment elevation myocardial infarction. Arch Cardiovasc Dis.

[14] Cannistraci CV, Nieminen T, Nishi M, Khachigian LM, Viikilä J, Laine M, Cianflone D, Maseri A, Yeo KK, Bhindi R, Ammirati E (2018). "Summer Shift": a potential effect of sunshine on the time onset of st-elevation acute myocardial infarction. J Am Heart Assoc.

[15] Anand SS, Islam S, Rosengren A, Franzosi MG, Steyn K, Yusufali AH, Keltai M, Diaz R, Rangarajan S, Yusuf S, INTERHEART Investigators (2008). Risk factors for myocardial infarction in women and men: insights from the INTERHEART study. Eur Heart J.

[16] Radišauskas R, Bernotienė G, Bacevičienė M, Ustinavičienė R, Kirvaitienė J, Krančiukaitė-Butylkinienė D (2014). Trends of myocardial infarction morbidity and its associations with weather conditions. Medicina (Kaunas).

[17] Stroup DF, Berlin JA, Morton SC, Olkin I, Williamson GD, Rennie D, Moher D, Becker BJ, Sipe TA, Thacker SB (2000). Meta-analysis of observational studies in epidemiology: a proposal for reporting. Meta-analysis of observational studies in epidemiology (MOOSE) group. JAMA.

[18] Kuzmenko NV, Tsyrlin VA, Pliss MG, Galagudza MM (2021). Seasonal variations in levels of human thyroid-stimulating hormone and thyroid hormones: a meta-analysis. Chronobiol Int.

[19] Borenstein M, Hedges LV, Higgins JP, Rothstein HR (2009). Introduction to meta-analysis.

[20] Depasquale NP, Burch GE (1961). The seasonal incidence of myocardial infarction in New Orleans. Am J Med Sci.

[21] Douglas AS, Dunnigan MG, Allan TM, Rawles JM (1995). Seasonal variation in coronary heart disease in Scotland. J Epidemiol Commun Health.

[22] González Hernández E, Cabadés O'Callaghan A, Cebrián Doménech J, López Merino V, Sanjuán Mañez R, Echánove Errazti I, Valencia Martín J, Bertomeu Martínez V, Investigadores del estudio PRIMVAC (2004). Variaciones estacionales en los ingresos por infarto agudo de micardio. El estudio PRIMVAC [Seasonal variations in admissions for acute myocardial infarction. The PRIMVAC study]. Rev Esp Cardiol.

[23] Heyer HE, Teng HC, Barris W (1953). The increased frequency of acute myocardial infarction during summer months in a warm climate; a study of 1,386 cases from Dallas, Texas. Am Heart J.

[24] Hong JS, Kang HC (2014). Seasonal variation in case fatality rate in Korean patients with acute myocardial infarction using the 1997–2006 Korean National Health insurance claims database. Acta Cardiol.

[25] Khan RC, Halder D (2014). Effect of seasonal variation on hospital admission due to cardiovascular disease - findings from an observational study in a divisional hospital in Bangladesh. BMC Cardiovasc Disord.

[26] Keller K, Hobohm L, Münzel T, Ostad MA (2019). Sex-specific differences regarding seasonal variations of incidence and mortality in patients with myocardial infarction in Germany. Int J Cardiol.

[27] Ku CS, Yang CY, Lee WJ, Chiang HT, Liu CP, Lin SL (1998). Absence of a seasonal variation in myocardial infarction onset in a region without temperature extremes. Cardiology.

[28] Lashari MN, Alam MT, Khan MS, Bawany FI, Qayoom M, Soomro K (2015). Variation in admission rates of acute coronary syndrome patients in coronary care unit according to different seasons. J Coll Physicians Surg Pak.

[29] Lin CC, Lee PY, Chen KC, Liao PC, Hsu JC, Li AH (2020). Clinical, demographic, and biochemical characteristics of patients with acute ST-segment elevation myocardial infarction: an analysis of acute coronary syndrome registry data of a Single Medical Center from 2005 to 2016. Acta Cardiol Sin.

[30] Mahajan AM, Gandhi H, Smilowitz NR, Roe MT, Hellkamp AS, Chiswell K, Gulati M, Reynolds HR (2019). Seasonal and circadian patterns of myocardial infarction by coronary artery disease status and sex in the ACTION Registry-GWTG. Int J Cardiol.

[31] Manfredini R, Manfredini F, Boari B, Bergami E, Mari E, Gamberini S, Salmi R, Gallerani M (2009). Seasonal and weekly patterns of hospital admissions for nonfatal and fatal myocardial infarction. Am J Emerg Med.

[32] Mintz SS, Katz LN (1947). Recent myocardial infarction: an analysis of five hundred and seventy-two cases. Arch Intern Med (Chic).

[33] Moschos N, Christoforaki M, Antonatos P (2004). Seasonal distribution of acute myocardial infarction and its relation to acute infections in a mild climate. Int J Cardiol.

[34] Nagarajan V, Fonarow GC, Ju C, Pencina M, Laskey WK, Maddox TM, Hernandez A, Bhatt DL (2017). Seasonal and circadian variations of acute myocardial infarction: findings from the get with the guidelines-coronary artery disease (GWTG-CAD) program. Am Heart J.

[35] Park IH, Jang WJ, Cho HK, Oh JH, Chun WJ, Park YH, Lee M, Song YB, Hahn JY, Choi SH, Lee SC, Gwon HC, Choe YH (2019). Season and myocardial injury in patients with ST-segment elevation myocardial infarction: a cardiac magnetic resonance imaging study. PLoS ONE.

[36] Rumana N, Kita Y, Turin TC, Murakami Y, Sugihara H, Morita Y, Tomioka N, Okayama A, Nakamura Y, Ueshima H (2008). Seasonal pattern of incidence and case fatality of acute myocardial infarction in a Japanese population (from the Takashima AMI Registry, 1988 to 2003). Am J Cardiol.

[37] Sharif Nia H, Gorgulu O, Pahlevan Sharif S, Froelicher ES, Haghdoost AA, Golshani S, Yaghoobzadeh A, Noble JH, Nazari R, Goudarzian AH, Arefinia F (2020). Prevalence of acute myocardial infarction and changing meteorological conditions in Iran: fuzzy clustering approach. Iran J Public Health.

[38] Spencer FA, Goldberg RJ, Becker RC, Gore JM (1998). Seasonal distribution of acute myocardial infarction in the second National Registry of Myocardial Infarction. J Am Coll Cardiol.

[39] Spielberg C, Falkenhahn D, Willich SN, Wegscheider K, Völler H (1996). Circadian, day-of-week, and seasonal variability in myocardial infarction: comparison between working and retired patients. Am Heart J.

[40] Thakur CP, Anand MP, Shahi MP (1987). Cold weather and myocardial infarction. Int J Cardiol.

[41] Vaičiulis V, Jaakkola JJK, Radišauskas R, Tamošiūnas A, Lukšienė D, Ryti NRI (2021). Association between winter cold spells and acute myocardial infarction in Lithuania 2000–2015. Sci Rep.

[42] Cheng J, Bambrick H, Tong S, Su H, Xu Z, Hu W (2020). Winter temperature and myocardial infarction in Brisbane, Australia: Spatial and temporal analyses. Sci Total Environ.

[43] Katayama Y, Tanaka A, Taruya A, Kashiwagi M, Nishiguchi T, Ozaki Y, Shiono Y, Shimamura K, Kitabata H, Kubo T, Hozumi T, Ishida Y, Kondo T, Akasaka T (2020). Increased plaque rupture forms peak incidence of acute myocardial infarction in winter. Int J Cardiol.

[44] Bhaskaran K, Hajat S, Haines A, Herrett E, Wilkinson P, Smeeth L (2010). Short term effects of temperature on risk of myocardial infarction in England and Wales: time series regression analysis of the Myocardial Ischaemia National Audit Project (MINAP) registry. BMJ.

[45] Kuzmenko NV, Tsyrlin VA, Pliss MG, Galagudza MM (2022). Seasonal fluctuations of blood pressure and heart rate in healthy people: a meta-analysis of panel studies. Hum Physiol.

[46] Shibuya J, Kobayashi N, Asai K, Tsurumi M, Shibata Y, Uchiyama S, Okazaki H, Goda H, Tani K, Shirakabe A, Takano M, Shimizu W (2019). Comparison of coronary culprit lesion morphology determined by optical coherence tomography and relation to outcomes in patients diagnosed with acute coronary syndrome during winter -vs- other seasons. Am J Cardiol.

[47] Kuzmenko NV, Pliss MG, Galagudza MM, Tsyrlin VA (2020). Effects of hyper- and hypothermia on hemodynamic parameters in people of different age groups: meta-analysis. Adv Gerontol.

[48] Gao Z, Wilson TE, Drew RC, Ettinger J, Monahan KD (2012). Altered coronary vascular control during cold stress in healthy older adults. Am J Physiol Heart Circ Physiol.

[49] Liu X, Kong D, Fu J, Zhang Y, Liu Y, Zhao Y, Lian H, Zhao X, Yang J, Fan Z (2018). Association between extreme temperature and acute myocardial infarction hospital admissions in Beijing, China: 2013–2016. PLoS ONE.

[50] Kuzmenko NV, Galagudza MM (2022). Dependence of seasonal dynamics of hemorrhagic and ischemic strokes on the climate of a region: a meta-analysis. Int J Stroke.

[51] Ginzburg AS, Vinogradova AA, Fedorova EI (2014). Content of oxygen in the atmosphere over large cities and respiratory problems. Izv Atmos Ocean Phys.

[52] Sharma S, Hashmi MF (2022) Partial Pressure Of Oxygen. In: StatPearls [Internet]. StatPearls Publishing, Treasure Island (FL) 29630271

[53] Goldberg MS, Giannetti N, Burnett RT, Mayo NE, Valois MF, Brophy JM (2008). A panel study in congestive heart failure to estimate the short-term effects from personal factors and environmental conditions on oxygen saturation and pulse rate. Occup Environ Med.

[54] Pope CA, Dockery DW, Kanner RE, Villegas GM, Schwartz J (1999). Oxygen saturation, pulse rate, and particulate air pollution: a daily time-series panel study. Am J Respir Crit Care Med.

[55] Gottlieb DJ, Yenokyan G, Newman AB, O'Connor GT, Punjabi NM, Quan SF (2010). Prospective study of obstructive sleep apnea and incident coronary heart disease and heart failure: the sleep heart health study. Circulation.

[56] Redline S, Yenokyan G, Gottlieb DJ, Shahar E, O'Connor GT, Resnick HE (2010). Obstructive sleep apnea hypopnea and incident stroke: the sleep heart health study. Am J Respir Crit Care Med.

[57] Klug G, Schenk S, Dörler J, Alber H, Mayr A, Schächinger V, Pachinger O, Metzler B (2011). Factors influencing the time-point of acute myocardial infarction in winter tourists. Int J Cardiol.

[58] Al Tahan A, Buchur J, el Khwsky F, Ogunniyi A, Al-Rajeh S, Larbi E, Daif A, Bamgboye E (1998). Risk factors of stroke at high and low altitude areas in Saudi Arabia. Arch Med Res.

[59] James SK, Erlinge D, Herlitz J, Alfredsson J, Koul S, Fröbert O, Kellerth T, Ravn-Fischer A, Alström P, Östlund O, Jernberg T, Lindahl B, Hofmann R, DETO2X-SWEDEHEART Investigators (2020). Effect of oxygen therapy on cardiovascular outcomes in relation to baseline oxygen saturation. JACC Cardiovasc Interv.

[60] Singhal AB (2006). Oxygen therapy in stroke: past, present, and future. Int J Stroke.

[61] Keatinge WR, Coleshaw SR, Easton JC, Cotter F, Mattock MB, Chelliah R (1986). Increased platelet and red cell counts, blood viscosity, and plasma cholesterol levels during heat stress, and mortality from coronary and cerebral thrombosis. Am J Med.

[62] Lavados PM, Olavarría VV, Hoffmeister L (2018). Ambient temperature and stroke risk: evidence supporting a short-term effect at a population level from acute environmental exposures. Stroke.

[63] Gagnon DR, Zhang TJ, Brand FN, Kannel WB (1994). Hematocrit and the risk of cardiovascular disease–the Framingham study: a 34-year follow-up. Am Heart J.

[64] Jin YZ, Zheng DH, Duan ZY, Lin YZ, Zhang XY, Wang JR, Han S, Wang GF, Zhang YJ (2015). Relationship between hematocrit level and cardiovascular risk factors in a community-based population. J Clin Lab Anal.

[65] Braekkan SK, Mathiesen EB, Njølstad I, Wilsgaard T, Hansen JB (2010). Hematocrit and risk of venous thromboembolism in a general population. The Tromso study. Haematologica.

[66] Ohki AV, van Bellen B (2017). A incidência regional do tromboembolismo venoso no Brasil. J Vasc Bras.

[67] Ruíz-Argüelles GJ, Sánchez-Medal L, Loría A, Piedras J, Córdova MS (1980). Red cell indices in normal adults residing at altitude from sea level to 2670 meters. Am J Hematol.

[68] Thorling EB, Erslev AJ (1968). The "tissue" tension of oxygen and its relation to hematocrit and erythropoiesis. Blood.

[69] Waltz X, Hardy-Dessources MD, Lemonne N, Mougenel D, Lalanne-Mistrih ML, Lamarre Y, Tarer V, Tressières B, Etienne-Julan M, Hue O, Connes P (2015). Is there a relationship between the hematocrit-to-viscosity ratio and microvascular oxygenation in brain and muscle?. Clin Hemorheol Microcirc.

[70] Brown HK, Simpson AJ, Murchison JT (2009). The influence of meteorological variables on the development of deep venous thrombosis. Thromb Haemost.

[71] Schobersberger W, Fries D, Mittermayr M, Innerhofer P, Sumann G, Schobersberger B, Klingler A, Stöllnberger V, Fischbach U, Gunga HC (2002). Changes of biochemical markers and functional tests for clot formation during long-haul flights. Thromb Res.

[72] Jang MJ, Kim HJ, Bang SM, Lee JO, Yhim HY, Kim YK, Kim YK, Choi WI, Lee EY, Kim IH, Park S, Sohn HJ, Kim DK, Kim M, Oh D (2012). Seasonal variation in the occurrence of venous thromboembolism: a report from the Korean Venous Thromboembolism working party. Thromb Res.

[73] Li Y, Ji C, Ju H, Han Y (2017). Impact of ambient temperature and atmospheric evaporation on the incidence of acute deep venous thrombosis in the northeast of China. Int Angiol.

[74] Ma QB, Zheng YA, Guo JX, Yao WZ, Li S, Wang Y, Dong XL (2011). Seven hundred and twenty-seven cases of acute pulmonary embolism and seasonal variation. Zhongguo Wei Zhong Bing Ji Jiu Yi Xue.

[75] Tan XY, He JG, Zou ZP, Zhao YF, Chen BP, Gao Y, Xiong CM, Ni XH, Cheng XS (2006). Changes of the proportion and mortality of pulmonary thromboembolism in hospitalized patients from 1974 to 2005. Chin Med J (Engl).

[76] Zhao H, Li Y, Wu M, Ren W, Ji C, Miao H, Han Y (2020). Seasonal variation in the frequency of venous thromboembolism: an updated result of a meta-analysis and systemic review. Phlebology.

[77] Mavri A, Guzic-Salobir B, Salobir-Pajnic B, Keber I, Stare J, Stegnar M (2001). Seasonal variation of some metabolic and haemostatic risk factors in subjects with and without coronary artery disease. Blood Coagul Fibrinolysis.

[78] Sung KC (2006). Seasonal variation of C-reactive protein in apparently healthy Koreans. Int J Cardiol.

[79] van der Bom JG, de Maat MP, Bots ML, Hofman A, Kluft C, Grobbee DE (1997). Seasonal variation in fibrinogen in the Rotterdam study. Thromb Haemost.

[80] Long B, Brady WJ, Koyfman A, Gottlieb M (2020). Cardiovascular complications in COVID-19. Am J Emerg Med.

[81] Warren-Gash C, Bhaskaran K, Hayward A, Leung GM, Lo SV, Wong CM, Ellis J, Pebody R, Smeeth L, Cowling BJ (2011). Circulating influenza virus, climatic factors, and acute myocardial infarction: a time series study in England and Wales and Hong Kong. J Infect Dis.

