# Supplementary material for: Seasonal dynamics of myocardial infarctions in regions with different types of a climate: a meta-analysis
Source: Egypt Heart J. 2022 Dec 22;74:84. doi: 10.1186/s43044-022-00322-5 (PMC9774076; doi:10.1186/s43044-022-00322-5)
Supplement: Supplementary file 1 — Additional file 1. Supplemental material including figures S1–S2 and tables S1–S2. [file 43044_2022_322_MOESM1_ESM.docx]

**SUPPLEMENTARY MATERIAL**

**Seasonal dynamics of myocardial infarctions in regions with different types of a climate: a meta-analysis**

N.V. Kuzmenko^1,а^, V.A. Tsyrlin^1^, M.G. Pliss^1^, M.M. Galagudza^1^

^1^ Department for Experimental Physiology and Pharmacology, Almazov National Medical Research Centre, 197341 St. Petersburg, Russia

**^a^Corresponding author: N.V. Kuzmenko, e-mail:** [**nat.kuzmencko2011@yandex.ru**](mailto:nat.kuzmencko2011@yandex.ru)

**CONTENT**

**Figure S1. Seasonal dynamics (in winter versus in summer) of MI risk (statistics, bias)……………………………..2**

**Figure S2. Seasonal dynamics (in spring versus in autumn) of MI risk (statistics, bias)………………………………5**

**Table S1. Quality score of the included studies………………………………………………………………………….8**

**Table S2. Justification for exclusion……………………………………………………………………………………….9**

**Figure S1. Seasonal dynamics (in winter versus in summer) of MI risk (statistics, bias)**

**All**

**
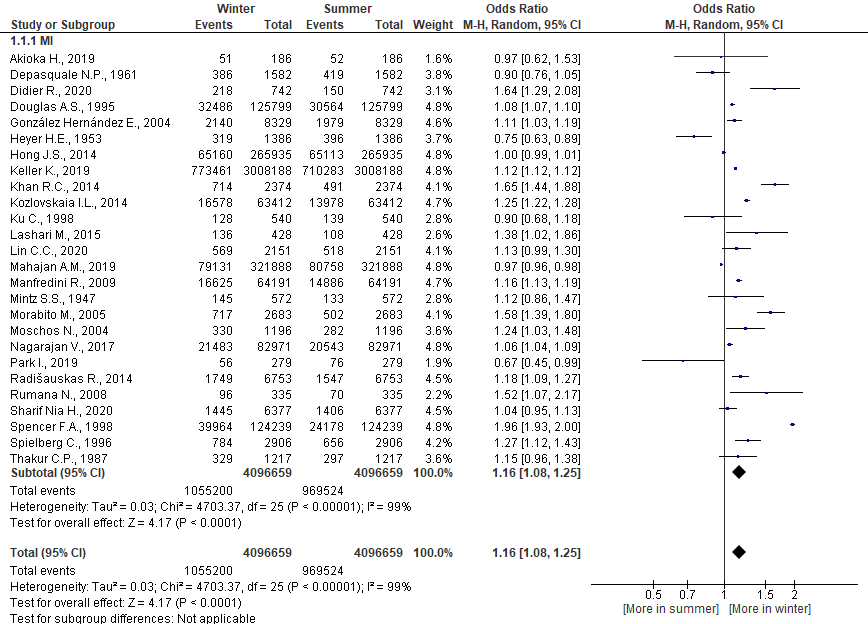
**


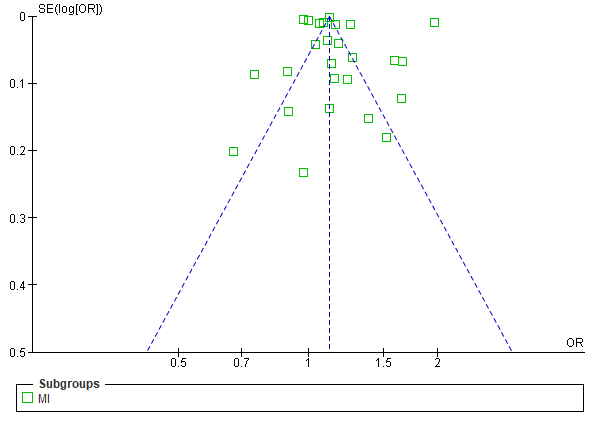


Egger's test P=0.63

Begg's test P=0.71

**Gender**

**
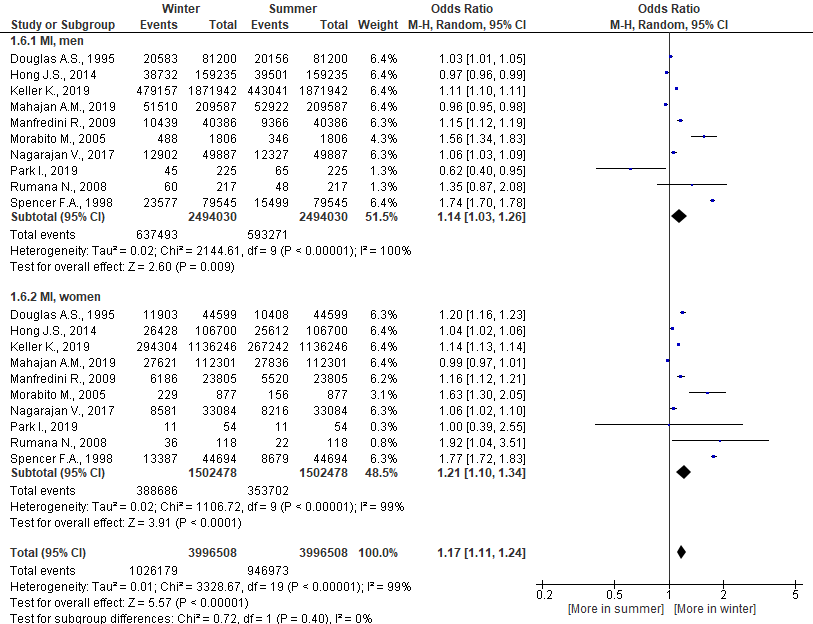
**

**
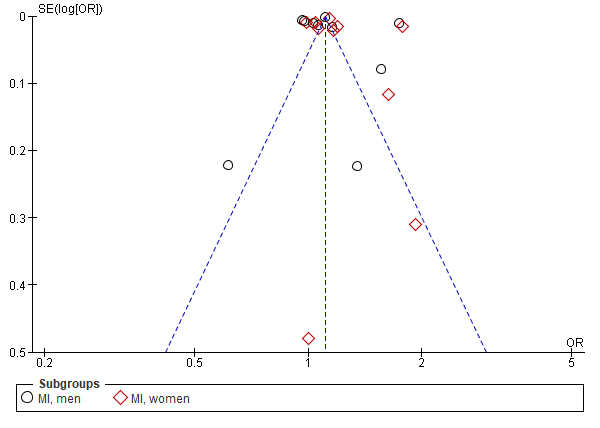
**

Egger's test - for men P=0.87 for women P=0.66

Begg's test - for men P=0.42 for women P=0.65

**Age**

**
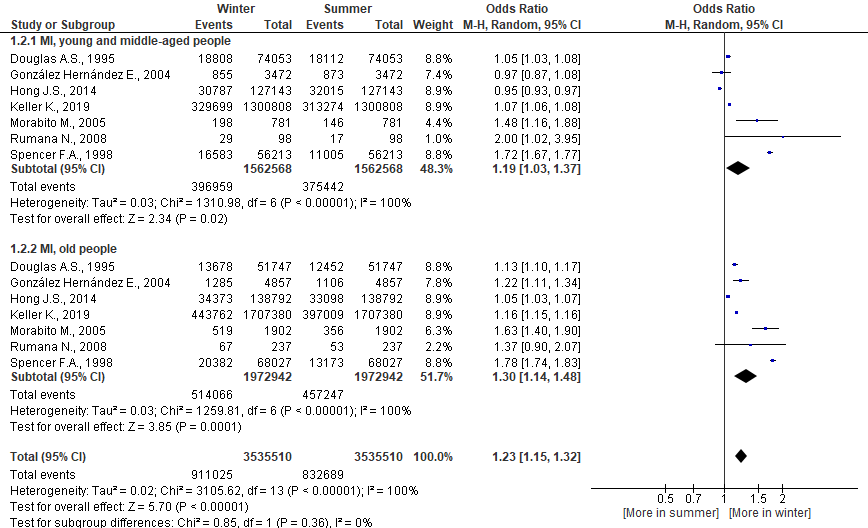
**

**
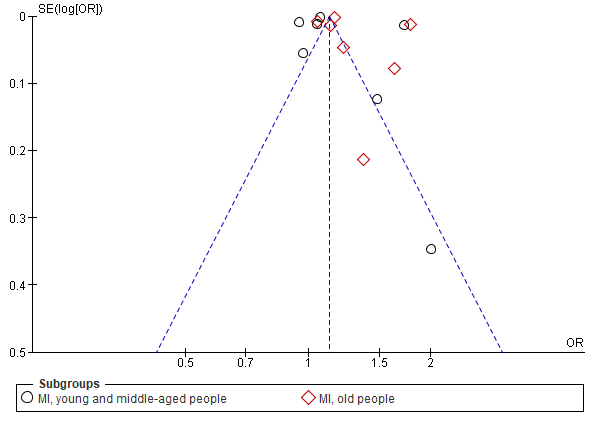
**

Egger's test - for young P=0.6 for old P=0.52

Begg's test - for young P=0.1 for old P=0.29

**Figure S2. Seasonal dynamics (in spring versus in autumn) of MI risk (statistics, bias)**

**All**

**
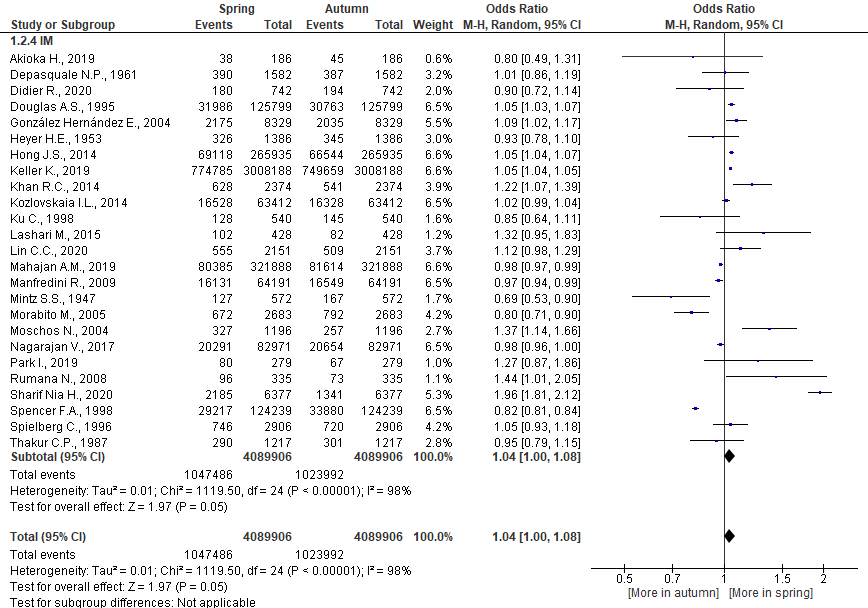
**

**
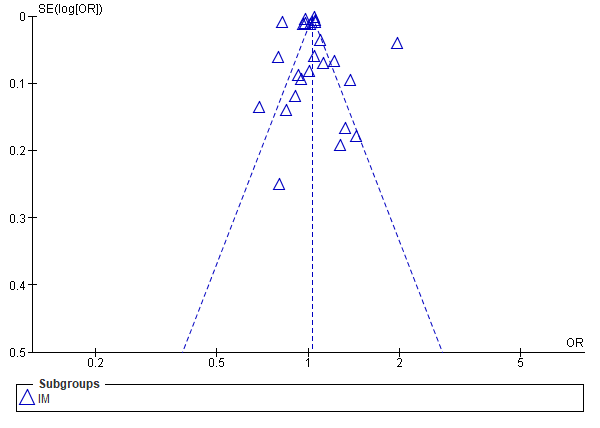
**

Egger's test P=0.61

Begg's test P=0.93

**Gender**

**
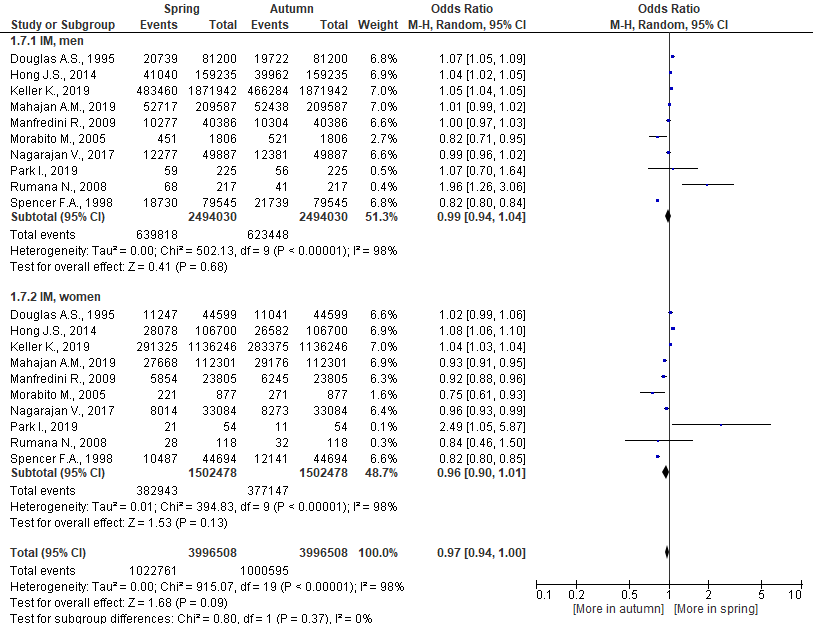
**

**
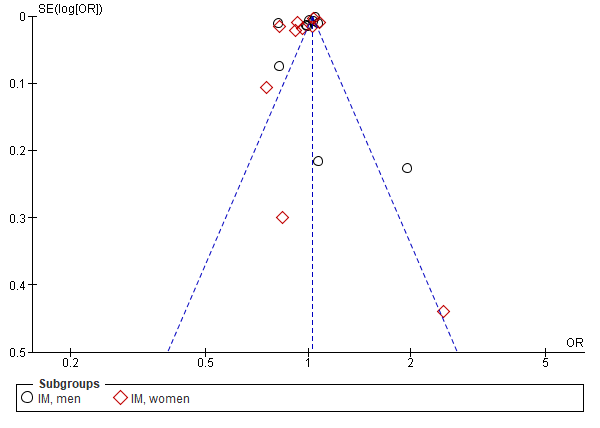
**

Egger's test - for men P=0.26 for women P=0.17

Begg's test - for men P=0.79 for women P=0.93

**Age**

**
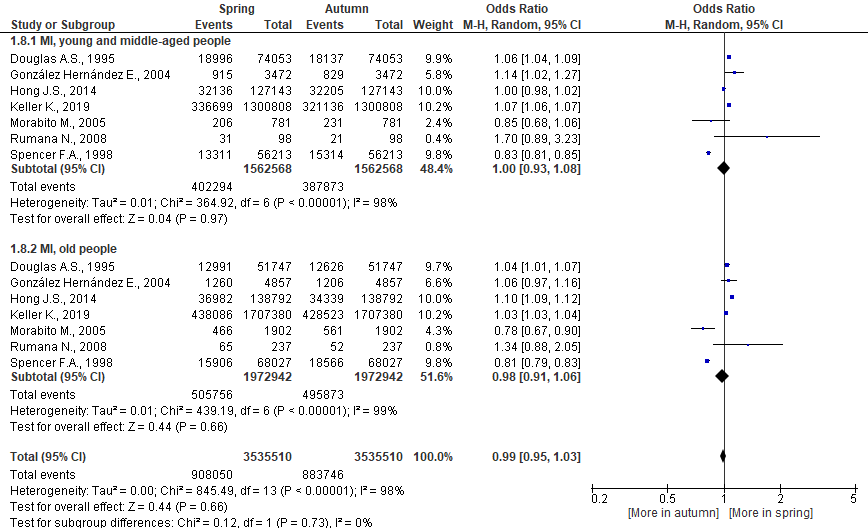
**

**
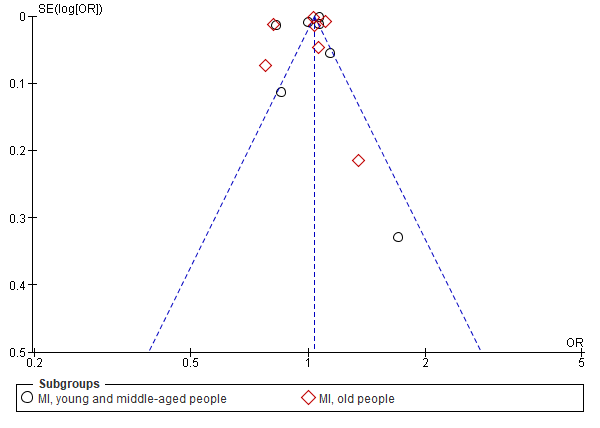
**

Egger's test - for young P=0.37 for old P=0.62

Begg's test - for young P=0.88 for old P=0.45

**Table S1. Quality score of the included studies**

| Publication | Diagnostics | Duration of observation | Study localization | Presentation of seasons | Presentation of meteorological data | Sample size  n>1000 | Gender.  data | Age data | Presentation of results | Points total |
| --- | --- | --- | --- | --- | --- | --- | --- | --- | --- | --- |
| Akioka Н., 2019 [8] | 0 | 1 | 2 | 2 | 2 | 0 | 2 | 2 | 2 | 13 |
| Depasquale N.P., 1961 [20] | 1 | 1 | 2 | 2 | 2 | 2 | 0 | 0 | 1 | 11 |
| Didier R., 2020 [13] | 1 | 2 | 2 | 2 | 1 | 0 | 2 | 2 | 2 | 14 |
| Douglas A.S., 1995 [21] | 0 | 2 | 0 | 2 | 0 | 2 | 2 | 2 | 2 | 16 |
| González Hernández E., 2004 [22] | 0 | 2 | 2 | 2 | 0 | 2 | 2 | 2 | 2 | 14 |
| Heyer H.E., 1953 [23] | 1 | 2 | 2 | 2 | 2 | 2 | 0 | 0 | 2 | 13 |
| Hong J.S., 2014 [24] | 0 | 2 | 0 | 2 | 1 | 2 | 2 | 2 | 2 | 13 |
| Keller K., 2019 [25] | 1 | 2 | 0 | 2 | 0 | 2 | 2 | 1 | 2 | 12 |
| Khan R.C., 2014 [26] | 2 | 1 | 2 | 2 | 1 | 2 | 0 | 0 | 2 | 12 |
| Kozlovskaia I.L., 2014 [9] | 1 | 1 | 2 | 2 | 1 | 2 | 2 | 2 | 1 | 14 |
| Ku С., 1998 [27] | 2 | 2 | 2 | 2 | 0 | 0 | 2 | 2 | 1 | 13 |
| Lashari М., 2015 [28] | 2 | 1 | 2 | 2 | 1 | 0 | 2 | 2 | 2 | 14 |
| Lin C.C., 2020 [29] | 0 | 2 | 2 | 2 | 0 | 2 | 2 | 2 | 2 | 14 |
| Mahajan A.M., 2019 [30] | 0 | 2 | 0 | 2 | 0 | 2 | 2 | 1 | 2 | 11 |
| Manfredini R., 2009 [31] | 2 | 2 | 2 | 2 | 0 | 2 | 2 | 2 | 2 | 16 |
| Mintz S.S., 1947 [32] | 1 | 2 | 2 | 2 | 0 | 0 | 2 | 2 | 2 | 13 |
| Morabito M., 2005 [11] | 2 | 2 | 2 | 2 | 0 | 2 | 2 | 0 | 1 | 13 |
| Moschos N., 2004 [33] | 2 | 2 | 2 | 2 | 1 | 2 | 2 | 2 | 2 | 17 |
| Nagarajan V., 2017 [34] | 0 | 2 | 0 | 2 | 0 | 2 | 2 | 2 | 2 | 12 |
| Park I., 2019 [35] | 2 | 2 | 2 | 2 | 0 | 0 | 2 | 2 | 2 | 14 |
| Radišauskas R., 2014 [16] | 2 | 2 | 2 | 1 | 1 | 2 | 0 | 2 | 1 | 13 |
| Rumana N., 2008 [36] | 2 | 2 | 2 | 2 | 0 | 0 | 2 | 2 | 2 | 14 |
| Sharif Nia H., 2020 [37] | 2 | 1 | 2 | 2 | 0 | 2 | 0 | 0 | 2 | 11 |
| Spencer F.A., 1998 [38] | 0 | 2 | 0 | 2 | 0 | 2 | 2 | 2 | 2 | 12 |
| Spielberg С., 1996 [39] | 2 | 2 | 2 | 2 | 1 | 2 | 2 | 2 | 1 | 16 |
| Thakur C.P., 1987 [40] | 2 | 2 | 2 | 2 | 1 | 2 | 0 | 0 | 2 | 13 |
| 0 is missing information, a small sample size. 1 is incomplete information, a small observation period. 2 is complete and well-presented information, a long observation period, a large sample size. | | | | | | | | | | |

**Table S2. Justification for exclusion**

| **№** | **Publication** | **Justification for exclusion** |
| --- | --- | --- |
|  | Sheth T, Nair C, Muller J, Yusuf S. Increased winter mortality from acute myocardial infarction and stroke: the effect of age. J Am Coll Cardiol. 1999 Jun;33(7):1916-9. doi: 10.1016/s0735-1097(99)00137-0. PMID: 10362193. | Mortality was investigated |
|  | Skajaa N, Horváth-Puhó E, Sundbøll J, Adelborg K, Rothman KJ, Sørensen HT. Forty-year Seasonality Trends in Occurrence of Myocardial Infarction, Ischemic Stroke, and Hemorrhagic Stroke. Epidemiology. 2018 Nov;29(6):777-783. doi: 10.1097/EDE.0000000000000892. PMID: 30028346. | Statistics |
|  | Mohammadian-Hafshejani A, Sarrafzadegan N, Hosseini S, Baradaran HR, Roohafza H, Sadeghi M, Asadi-Lari M. Seasonal pattern in admissions and mortality from acute myocardial infarction in elderly patients in Isfahan, Iran. ARYA Atheroscler. 2014 Jan;10(1):46-54. PMID: 24963314; PMCID: PMC4063513. | Mountain climate |
|  | Çatalkaya Demir S, Demir E, Çatalkaya S. Electrocardiographic and Seasonal Patterns Allow Accurate Differentiation of Tako-Tsubo Cardiomyopathy from Acute Anterior Myocardial Infarction: Results of a Multicenter Study and Systematic Overview of Available Studies. Biomolecules. 2019 Jan 30;9(2):51. doi: 10.3390/biom9020051. PMID: 30704132; PMCID: PMC6406531. | Statistics |
|  | Harvey NC, Lorentzon M, Kanis JA, McCloskey E, Johansson H. Incidence of myocardial infarction and associated mortality varies by latitude and season: findings from a Swedish Registry Study. J Public Health (Oxf). 2019 Nov 28:fdz131. doi: 10.1093/pubmed/fdz131. Epub ahead of print. PMID: 31774530. | Statistics |
|  | Hodzic E, Perla S, Iglica A, Vucijak M. Seasonal Incidence of Acute Coronary Syndrome and Its Features. Mater Sociomed. 2018 Mar;30(1):10-14. doi: 10.5455/msm.2018.30.10-14. PMID: 29670472; PMCID: PMC5857057. | Statistics (only 2 seasons) |
|  | Yang J, Zhou M, Ou CQ, Yin P, Li M, Tong S, Gasparrini A, Liu X, Li J, Cao L, Wu H, Liu Q. Seasonal variations of temperature-related mortality burden from cardiovascular disease and myocardial infarction in China. Environ Pollut. 2017 May;224:400-406. doi: 10.1016/j.envpol.2017.02.020. Epub 2017 Feb 20. PMID: 28222981. | Mortality was investigated |
|  | Liu X, Bertazzon S. Exploratory Temporal and Spatial Analysis of Myocardial Infarction Hospitalizations in Calgary, Canada. Int J Environ Res Public Health. 2017 Dec 11;14(12):1555. doi: 10.3390/ijerph14121555. PMID: 29232910; PMCID: PMC5750973. | Mountain climate |
|  | Houck PD, Lethen JE, Riggs MW, Gantt DS, Dehmer GJ. Relation of atmospheric pressure changes and the occurrences of acute myocardial infarction and stroke. Am J Cardiol. 2005 Jul 1;96(1):45-51. doi: 10.1016/j.amjcard.2005.02.042. PMID: 15979431. | Statistics (graphics are difficult to interpret) |
|  | Keller K, Hobohm L, Münzel T, Ostad MA. Sex-specific differences regarding seasonal variations of incidence and mortality in patients with myocardial infarction in Germany. Int J Cardiol. 2019 Jul 15;287:132-138. doi: 10.1016/j.ijcard.2019.04.035. Epub 2019 Apr 11. PMID: 31005418. | Mortality was investigated |
|  | Rivero A, Bolufé J, Ortiz PL, Rodríguez Y, Reyes MC. Influence of climate variability on acute myocardial infarction mortality in Havana, 2001-2012. MEDICC Rev. 2015 Apr;17(2):14-9. PMID: 26027582. | Mortality was investigated |
|  | Ornato JP, Peberdy MA, Chandra NC, Bush DE. Seasonal pattern of acute myocardial infarction in the National Registry of Myocardial Infarction. J Am Coll Cardiol. 1996 Dec;28(7):1684-8. doi: 10.1016/s0735-1097(96)00411-1. PMID: 8962552 | Statistics |
|  | Warren-Gash C, Bhaskaran K, Hayward A, Leung GM, Lo SV, Wong CM, Ellis J, Pebody R, Smeeth L, Cowling BJ. Circulating influenza virus, climatic factors, and acute myocardial infarction: a time series study in England and Wales and Hong Kong. J Infect Dis. 2011 Jun 15;203(12):1710-8. doi: 10.1093/infdis/jir171. PMID: 21606529; PMCID: PMC3100509. | Statistics (graphics are difficult to interpret) |
|  | Dilaveris P, Synetos A, Giannopoulos G, Gialafos E, Pantazis A, Stefanadis C. CLimate Impacts on Myocardial infarction deaths in the Athens TErritory: the CLIMATE study. Heart. 2006 Dec;92(12):1747-51. doi: 10.1136/hrt.2006.091884. Epub 2006 Jul 13. PMID: 16840509; PMCID: PMC1861268. | Mortality was investigated |
|  | Gerber Y, Jacobsen SJ, Killian JM, Weston SA, Roger VL. Seasonality and daily weather conditions in relation to myocardial infarction and sudden cardiac death in Olmsted County, Minnesota, 1979 to 2002. J Am Coll Cardiol. 2006 Jul 18;48(2):287-92. doi: 10.1016/j.jacc.2006.02.065 | Statistics |
|  | Shibuya J, Kobayashi N, Asai K, Tsurumi M, Shibata Y, Uchiyama S, Okazaki H, Goda H, Tani K, Shirakabe A, Takano M, Shimizu W. Comparison of Coronary Culprit Lesion Morphology Determined by Optical Coherence Tomography and Relation to Outcomes in Patients Diagnosed with Acute Coronary Syndrome During Winter -vs- Other Seasons. Am J Cardiol. 2019 Jul 1;124(1):31-38. doi: 10.1016/j.amjcard.2019.03.045 | Season |
|  | Shih CY, Chu ML, Hsieh TC, Chen HL, Lee CW. Acute Myocardial Infarction among Young Adult Men in a Region with Warm Climate: Clinical Characteristics and Seasonal Distribution. Int J Environ Res Public Health. 2020 Aug 24;17(17):6140. doi: 10.3390/ijerph17176140 | Only men were studied |
|  | da Silva GAP, Kock KS. Effect of seasonality in hospitalizations and deaths from acute myocardial infarction in southern Brazil from 2009 to 2018. Am J Cardiovasc Dis. 2021 Feb 15;11(1):148-154. PMID: 33815930; PMCID: PMC8012281. | Statistics |
|  | Vaičiulis V, Jaakkola JJK, Radišauskas R, Tamošiūnas A, Lukšienė D, Ryti NRI. Association between winter cold spells and acute myocardial infarction in Lithuania 2000-2015. Sci Rep. 2021 Aug 23;11(1):17062. doi: 10.1038/s41598-021-96366-9. | Statistics |
|  | Sakelliadis EI, Katsos KD, Zouzia EI, Vlachodimitropoulos DG, Goutas ND, Spiliopoulou CA. Biological rhythms of fatal myocardial infarction in Greece: an autopsy study. Acta Cardiol. 2021 Dec;76(10):1092-1099. doi: 10.1080/00015385.2020.1834248. | Mortality was investigated |
